# Fatal non-thrombotic pulmonary embolization in a patient with undiagnosed factitious disorder

**DOI:** 10.1186/s13104-015-1265-y

**Published:** 2015-07-12

**Authors:** Younghoon Kwon, Ryan J Koene, Caroline Cross, Jennifer McEntee, Jaime S Green

**Affiliations:** Department of Medicine, University of Minnesota, 420 Delaware Street S.E., Minneapolis, MN 55455 USA; Department of Laboratory Medicine and Pathology, University of Minnesota, Minneapolis, MN USA; Department of Medicine, Duke University, Durham, NC USA

**Keywords:** Fever of unknown origin, Microcrystalline cellulose emboli, Catheter-associated bloodstream infections, Factitious disorder

## Abstract

**Background:**

Factitious fever is extremely challenging to diagnose in patients with complicated chronic medical problems, and represents as much as 10% of fevers of unknown origin. Factitious fever caused by self-injecting oral medications through indwelling central catheters is a diagnostic challenge.

**Case presentation:**

We present a 32-year-old Caucasian female with history of short gut syndrome, malnutrition requiring total parental nutrition, and pancreatic auto-islet transplant with fever of unknown origin. Multiple episodes of bacteremia occurred with atypical pathogens, including α-hemolytic Streptococcus, *Achromobacter xylosoxidans*, and *Mycobacterium mucogenicum*. Chest computed tomography was notable for extensive tree-in-bud infiltrates. Sudden cardiac arrest with right-sided heart failure following acute hypoxemia led to her death. Diffuse microcrystalline cellulose emboli with foreign body granulomatosis was found on autopsy. Circumstantial evidence indicated that this patient suffered from factitious disorder, and was self-injecting oral medications through her central catheter.

**Conclusion:**

A high index of suspicion, early recognition, and multifaceted team support is essential to detect and manage patients with factitious disorders before fatal events occur.

## Background

Fever of unknown origin (FUO) is defined as a fever for 3 or more weeks with a diagnosis that remains uncertain after 1 week of investigation. Due to improved diagnostic testing over several decades infectious and neoplastic causes of FUO have declined while unidentified causes have increased [1]. Factitious fever represents 1–10% of FUO cases [2, 3]. The most comprehensive review of factitious fevers, published in 1979, included 32 cases [3]. Polymicrobial bacteremia from self-injection occurred in five subjects from this study, and has also been reported in other sporadic case reports [4, 5]. All of these studies were published prior to the widespread use of long-term central line catheters, which are routinely used in clinical practice today. Central line-related bloodstream infections are responsible for 40% of healthcare-associated infection [6]. The impact of long-term central line catheters on self-inflicting harmful behaviors, such as factitious disorders, is difficult to quantitate, and recognition of these disorders remains an ongoing challenge for healthcare providers.

## Case report

We present a 32-year-old Caucasian female with a past medical history of pseudotumor cerebri (ventriculoperitoneal shunt in place) and morbid obesity managed with Roux-en-Y gastric bypass. Two years after gastric bypass, necrosis of the distal pouch, stomach, and duodenum occurred, necessitating surgical debridement with abdominal reconstruction. She subsequently developed chronic malnutrition and short gut syndrome requiring long-term total parenteral nutrition through a peripherally inserted central catheter (PICC). Due to ongoing chronic pancreatitis, a pancreatic-auto-islet cell transplant with splenectomy was performed.

Five months following the pancreatic-auto-islet cell transplant, she was hospitalized three times during the same month with fever and abdominal pain. *Hospitalization one* Fevers were documented up to 101.2°F with a mild leukocytosis (11,400 cells/mm^3^). Blood cultures were positive for α-hemolytic Streptococcus (Table 1). The PICC line was removed and replaced before discharge. *Hospitalization two* Fevers were documented up to 100.8°F with a leukocytosis of 21,000 cells/mm^3^. Blood cultures were negative. Computed tomography (CT) imaging of the abdomen and pelvis did not demonstrate a source for the fever or leukocytosis. Nutritional support was maintained through enteric tube feeds, and at the patient’s preference a PICC line was again replaced for daily fluid infusion. *Hospitalization three* Fevers were documented up to 103°F for 3 days, with a leukocytosis of 14,300 cells/mm^3^. Blood cultures from the PICC line and peripheral draw grew α-hemolytic Streptococcus and *Achromobacter* spp. on two consecutive days. No fluid collections or intra-abdominal abscesses were found on CT imaging of the abdomen and pelvis. A chest radiograph and transthoracic echocardiogram were normal. The α-hemolytic Streptococcus and *Achromobacter* spp. (Table 1) were treated with 14 days of piperacillin–tazobactam.

**Table 1:** Microbiologic data

	Hospitalization 1	Hospitalization 2	Hospitalization 3	Hospitalization 4
Temperature	101.2°F	100.8°F	103°F	Afebrile
Blood cultures	Peripherally inserted central catheter: α-hemolytic Streptococcus (+)	No growth	Peripherally inserted central catheter and peripheral intravenous blood collection: α-hemolytic Streptococcus and *Achromobacter xylosoxidans* (+)	Peripherally inserted central catheter: *Mycobacterium mucogenicum* (+)
Viral studies				Polymerase chain reaction blood (−) for Adenovirus, cytomegalovirus, and Ebstein–Barr virus. Bronchoalveolar lavage (−) for influenza and respiratory syncytial virus
Fungal studies				Histoplasma and blastomyces antigen urine (−). Cryptococcal antigen blood (−)

**Figure 1:**
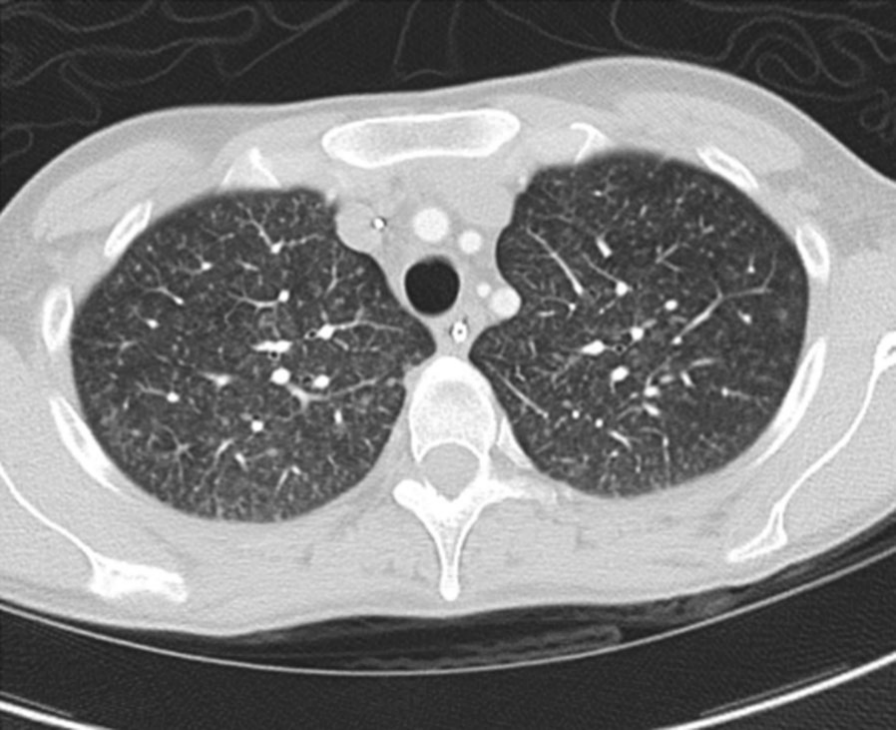
High resolution chest computed tomography scan. Centrilobular nodules in the tree-in-bud configuration.

In clinic, following the third hospitalization, this patient reported daily fevers up to 104°F that started 1 day after being discharged from the hospital (while on piperacillin–tazobactam). She reported head and neck pain with a sore throat, which increased in severity during the febrile episodes. Mild fever of 100.9°F was present. Physical exam was normal, including the oropharynx and skin exit site of the PICC line. Outpatient workup included blood cultures, viral studies, and fungal blood tests (all were negative) (Table 1). High-resolution chest CT revealed extensive tree-in-bud infiltrates (Figure 1). The following day she reported high fevers and was admitted to the hospital for an expedited work up.

**Figure 2:**
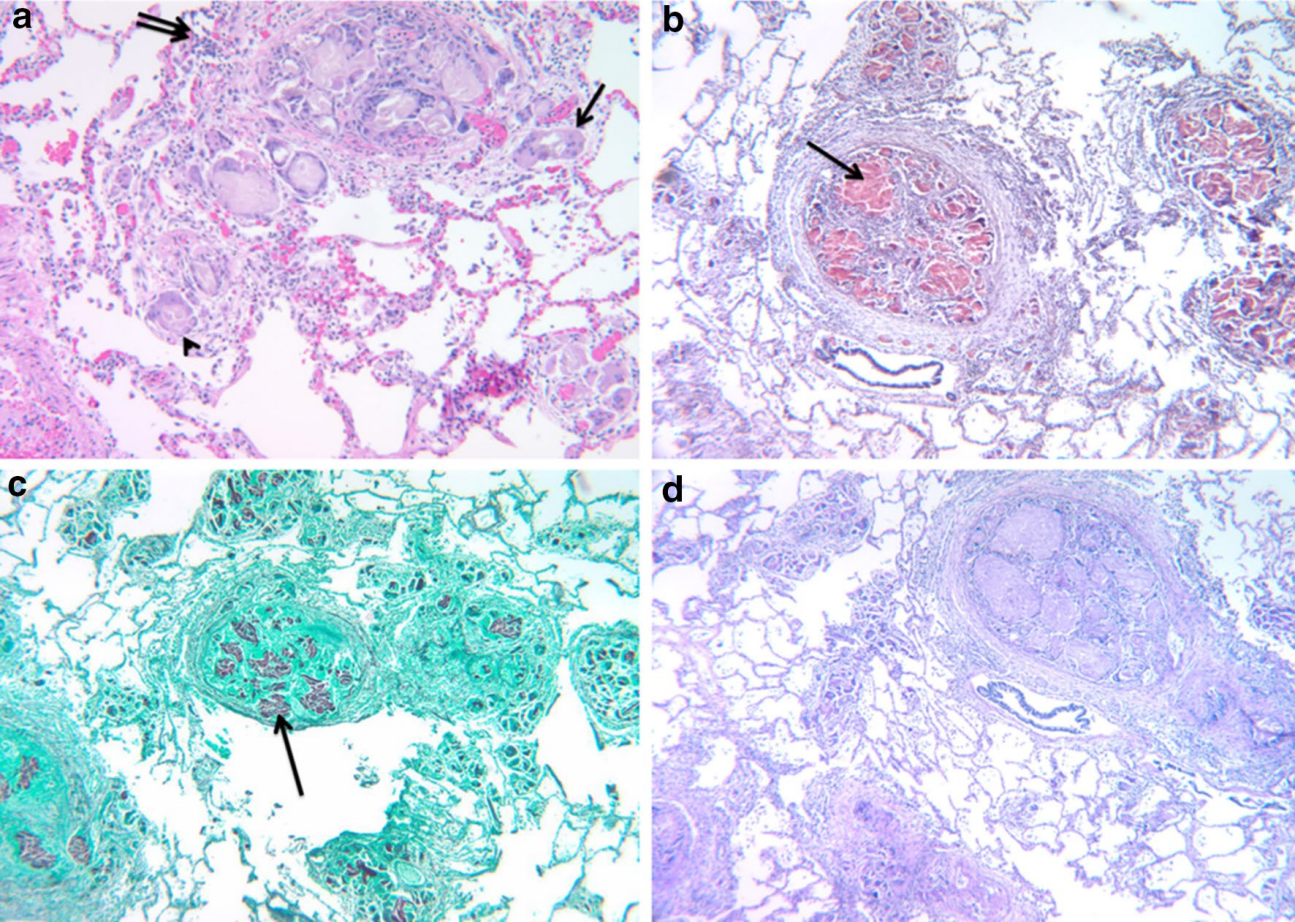
**a** Microcrystalline cellulose embolization affecting arterioles (*arrowhead*) and medium-sized pulmonary arteries (*top-center*) with associated perivascular granulomatous reaction consisting of multi-nucleated giant cells (*long arrow*) and epithelioid histiocytes (*double long arrow*) (Hematoxylin & Eosin, original magnification ×100). **b** Microcrystalline cellulose fibers stained *salmon pink* (Congo red, original magnification ×400). **c** Microcrystalline cellulose fibers stained *gray-black* with Gomori methenamine-silver (original magnification ×400). **d** Microcrystalline cellulose stained magenta with PAS (periodic Acid-Schiff stain, original magnification ×400).

During *hospitalization four* there were no documented fevers. Bronchoalveolar lavage was non-diagnostic with negative cultures and cytology. The morning of hospital day five, the patient developed acute chest pain, dyspnea, mild hypotension, and hypoxia. Intravenous fluid and oxygen supplementation were administered. Echocardiogram showed new marked right ventricular dilatation with severe right ventricular dysfunction. Due to concerns for pulmonary emboli (PE), intravenous heparin therapy was initiated. CT pulmonary angiogram was negative for PE. That afternoon she was found unresponsive in pulseless electrical activity arrest. The patient died despite extensive resuscitation efforts. *Mycobacterium mucogenicum* eventually grew from the PICC line culture drawn from hospital day one. The cause of death at autopsy was attributed to complications from pulmonary microcrystalline cellulose emboli with associated foreign body granulomatosis (Figure 2).

## Discussion

This case demonstrates the challenge of recognizing factitious disorders in complicated medical patients with chronic conditions. The FUO with recurrent bacteremia due to atypical pathogens likely resulted from self-administration of oral medications through the center catheter, which ultimately led to a fatal pulmonary complication of microcrystalline cellulose and foreign body granulomatosis.

Recurrent polymicrobial bacteremia with an unknown source must raise suspicion of self-inflicted illness. Our patient was asplenic with a history of multiple abdominal surgeries and pancreatic-auto-islet transplant. There were many legitimate reasons to have an organic cause of fevers for which she was extensively evaluated. However, it was not until autopsy that it became clear that our patient was self-injecting oral medications through the central catheter.

Both malingering syndrome and factitious disorders misdirect practitioners, often resulting in exhaustive medical evaluations to identify organic causes of the fever. The motivating factors (either primary or secondary gain) distinguish the two conditions. Adult patients are more sophisticated in masking medical conditions, and often involve subjects with some medical background. In a 10-year review of 41 patients with factitious disorders, 39 were female and 28 held medical jobs. Once confronted with evidence that their symptoms were self-induced, many illnesses resolved or improved (17/33 cases) [4]. The mortality rate from factitious disorders ranges from 2 to 20% in small case series, [7–9] but is likely underrepresented and under diagnosed.

In this case, the isolation of atypical organisms, (α-hemolytic Streptococcus, *M. mucogenicum,* and *Achromobacter* spp.) is consistent with administration of unsterile solutions through the central line. Alpha-hemolytic Streptococcus is a commensal organism found in the oral flora. *M. mucogenicum* and *Achromobacter* spp. naturally reside in water and soil. *M. mucogenicum* is a rapidly growing mycobacterium with a highly mucoid character on solid agar [10], which may contribute to the pathogenesis of catheter infections [11]. In a large series of rapidly growing mycobacteria infections, *M. mucogenicum* represented the most common cause of catheter and bloodstream infections (24 of 46 cases) among a population of cancer and immunocompromised patients [12]. Bloodstream infection with *M. mucogenicum* has been associated with dialysis catheters, and water-borne central line infections [13, 14]. Kline et al., from our hospital, reported an outbreak of *M. mucogenicum* associated catheter infections from hospital and clinic tap water that contaminated the catheters during routine care and bathing [15].

*Achromobacter* spp. is considered an emerging pathogen in catheter related infections, and has been reported with contaminated dialysis fluid, chlorhexidine solutions, and mechanical ventilators [16, 17]. Two large series of *Achromobacter* spp. bloodstream infections have been published. In a cohort of cancer patients, 47 episodes of *Achromobacter* spp. bacteremia were characterized. Twenty-five percent were catheter associated, and 33% were nosocomially acquired [18]. Gomez-Cerezo et al. described 54 cases of *Achromobacter* spp. bacteremia over 10 years, in which 65% were considered catheter-related. During that time, an epidemic outbreak occurred in the hemodialysis unit. Transmission was thought to occur from healthcare workers hands after washing with contaminated hospital water [19].

Ultimately, our patient died from extensive pulmonary granulomatosis caused by the deposition of microcrystalline cellulose in the pulmonary tree with non-thrombotic pulmonary emboli (NTPE), right-sided heart failure, and sudden cardiac arrest. Microcrystalline cellulose is an inactive component commonly used as filler for oral medications including methadone and hydromorphone. With inappropriate intravenous administration, the crushed insoluble component of the oral medication (microcrystalline cellulose in our case) becomes trapped in the pulmonary arterioles causing an inflammatory cascade, thrombosis, and formation of granulomas [20]. Widespread pulmonary granulomatosis has been described in long term intravenous drug abusers, [20] and patients with factitious disorders [21]. Intravenous administration of illicit drugs, or drugs intended for oral administration, have been linked to foreign body-related NTPE [22, 23]. Foreign body embolization to pulmonary circulation can remain asymptomatic for years [24] or can be acutely fatal as demonstrated in our patient. The incidence of fatal NTPE related to intravenous administration of oral medication might be higher than reported, since diagnosis is often made on post mortem examination.

The fevers that occurred in our patient were factitious. Our patient first learned to crush her pills to administer them through the gastric-jejunal tube. In hindsight, it is logical that our patient later began to administer her oral crushed medications through the central catheter in an attempt to manage her chronic pain.

## Conclusions

Polymicrobial and recurrent bacteremia with unusual organisms in the setting of long term indwelling central lines must alert physicians to consider self-injection of unsterile materials and factitious disorders. Recognition of these conditions becomes even more difficult in patients with complex medical conditions. In the modern era of medicine, where care may be fragmented with lack of continuity, the diagnosis of factitious syndromes and misuse of central lines are even more challenging. As this case demonstrates, factitious disorders can be fatal. It is essential that health care providers maintain a high index of suspicion of self-harming behaviors so that these syndromes can be recognized early. A multidisciplinary team approach can implement care plans to improve the outcome in these patients.

## Abbreviations

CT: computerized tomography
FUO: fever of unknown origin
NTPE: non-thrombotic pulmonary emboli
PICC: peripherally inserted central catheter
